# Supplementary material for: Informing agent-based models with spatial data using convolutional autoencoders
Source: Bioinformatics. 2026 Aug 21;42(Suppl 2):btag444. doi: 10.1093/bioinformatics/btag444 (PMC13501296; doi:10.1093/bioinformatics/btag444)
Supplement: Supplementary file 1 [file btag444_supplementary_data.pdf]

Supplementary Information

Informing agent-based models with spatial data using convolutional autoencoders

Authors
Wang B.[ab]
Liao C.[c]
Danen E.H.J.[c]
Neubert E.[c]
Eduati F.*[ab]

*=corresponding author

Author affiliations
[a]Department of Biomedical Engineering, Eindhoven University of Technology, PO Box 513, Eindhoven 5600MB, the Netherlands

[b]Institute for Complex Molecular Systems, Eindhoven University of Technology, 5600 MB Eindhoven, The Netherlands

[c]Leiden Academic Center for Drug Research, Leiden University, Leiden, the Netherlands

Contact information
Lead contact: Eduati F. (f.eduati@tue.nl)

**Supplementary Table: Parameter values for different data sets**<br>*(*) Optimized parameter, range is given instead of fixed value (range defined on a combination of preliminary simulation resulting in realistic outputs and biological plausibility)*<br>*[1]Fixed value chosen based on preliminary simulations to achieve outputs representing experimental setup and computational feasibility. For several cases where this value differed across data sets, a small elaboration will be provided.*<br>*[2]Fixed value based on properties from experimental setup*<br>*[3]Fixed value based on literature value from Kather et al. (2018)*				
**Parameter**	**Description**	**Synthetic**	**Tumoroid**	**TCGA**
**oneStepDuration**	Time interval for each simulation step	12[3]	1[2] (one frame in simulation could then correspond with one frame from timelapse)	12[3]
**Height, width**	Height and width (number of cells) within simulation grid	100[1] (allowed for variation across parameter ranges (e.g. both spheroids and dense grids could be achieved) while remaining computationally feasible)	100[1]	25[1] (decreased so larger patches of tumor and lymphocytes can be simulated)
**nSteps**	Number of steps per simulation	150[1] (based on achieving a balance between reduced variation across simulations and computational feasibility, also still allowed for both spheroid and completely filled grid simulations as longer simulations resulted in all spheroids	72[2] (timelapses were 72 hours, same reasoning as for oneStepDuration)	100[1]

		reaching the edge of the grid)		
**TUpprol**	Tumor proliferation probability	(*) 0.1-0.5	0.15[1]	(*) 0.1 - 1
**TUpmig**	Tumor migration probability	0.05[1]	0.0005[1] *(lower TUpmig because tumoroids retained their spherical morphology during experiments, else too much single tumor cell dissociation)*	0.05[1]
**TUpdeath**	Tumor death probability	0.05[1]	0.05[1]	0.05[1]
**TUpmax**	Maximum number of times tumor can proliferate	10[3]	10[3]	10[3]
**TUdamageThresh**	How much damage tumor requires before lymphocyte successfully killed the cell	2[3]	2[3]	2[3]
**IMkmax**	Maximum number of times lymphocyte can kill before becoming exhausted	15[1]	15[1]	15[1]
**IMpmax**	Maximum number of times lymphocyte can proliferate	5[3]	5[3]	5[3]
**IMpmig**	Lymphocyte migration probability	0.8[3]	0.9[1] *(a lower migration probability often resulted in limited movement due nSteps being smaller than other two data sets)*	0.8[3]
**IMpkill**	Lymphocyte killing probability	(*) 0.1 - 0.8	(*) 0.1 - 0.8	(*) 0.1 - 1
**IMpprol**	Lymphocyte proliferation probability	4.6289e-4[3]	4.6289e-4[3]	4.6289e-4[3]

**IMpdeath**	Lymphocyte death probability	1.5173e-4[3]	0.2[1] *(indirect approach to mimic lymphocytes travelling across Z-stacks, else simulations resulted in large numbers of lymphocytes not representative of the tumoroid data)*	1.5173e-4[3]
**IMinfluxProb**	Lymphocyte influx probability	0.1[1]	0.2[1] *(to mimic lymphocytes traveling across Z-stacks)*	(*) 0.1 - 1
**IMinfluxRate**	Number of lymphocytes that enter simulation during influx	1[3]	1[3]	2[1] *(to make it more feasible to achieve the dense areas of lymphocytes observed in the TCGA patches)*
**IMrateDynamic**	Scaling parameter to increase number of lymphocytes entering simulation during influx w.r.t. tumor cells	0.0075[3]	0.0075[3]	0.0075[3]
**IMrwalk**	Lymphocyte random walk probability to distinguish between directed and random migration	(*) 0 - 1	0[1] *(especially with limited nSteps, if not completely directed, lymphocytes were not able to reach the tumoroid consistently, making it challenging to analyze killing efficiency)*	0.5[1]
**IMinfluxEdge**	Whether lymphocyte influx can only occur at edge of simulation grid	True[1] *(assumption that influx*	False[2] *(based on lymphocytes being able to travel*	False[2] *(based on the data being patches*

	(True) or at random throughout entire grid (False)	*occurs from blood vessel (so from edge))*	*across Z-stacks, from where they can appear anywhere in the grid)*	*from anywhere in the tumor)*

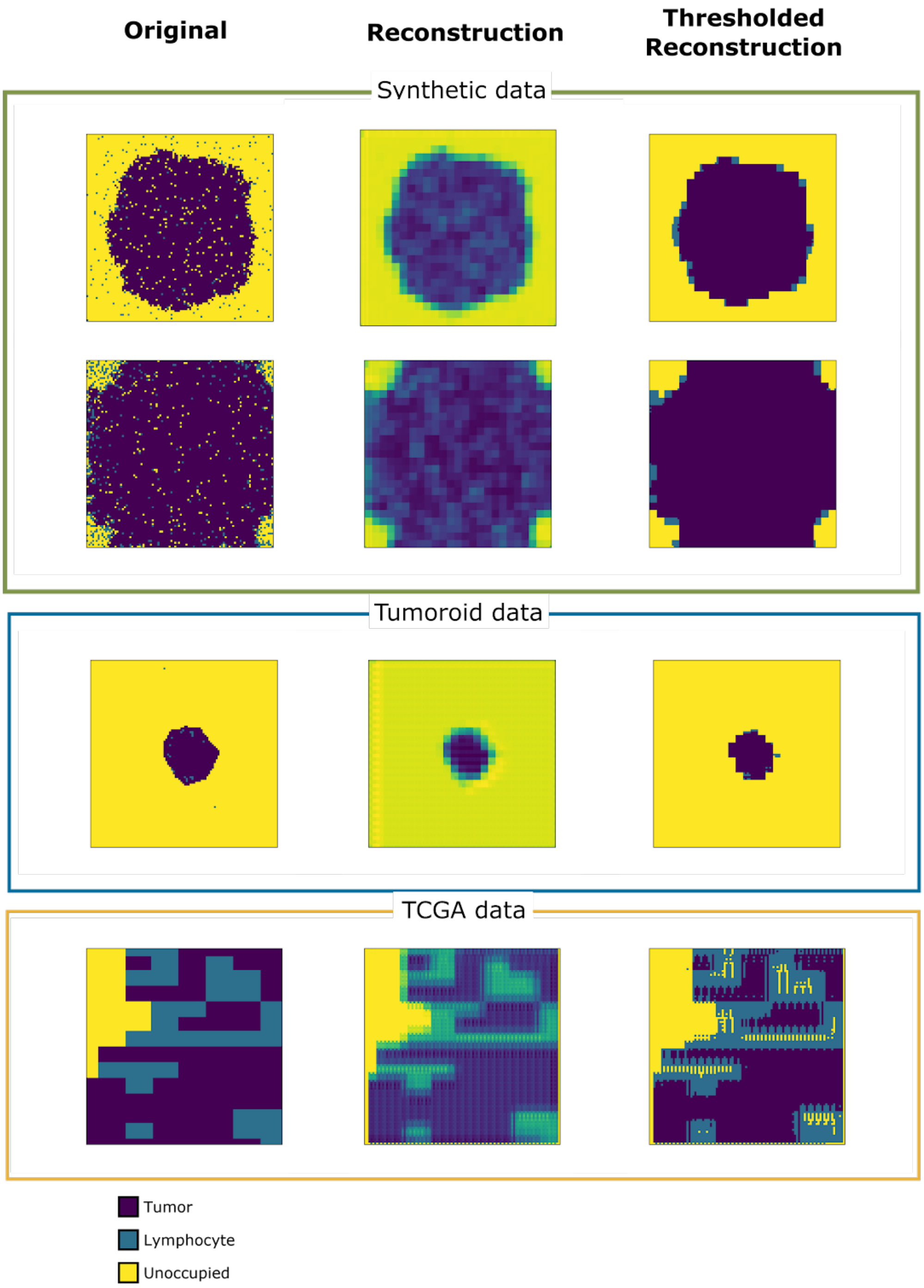

Supplementary Figure 1: Reconstruction of images with trained autoencoder. Examples are visualized for different data sets, including original and reconstructed (by trained autoencoder) image and a thresholded reconstruction image. The empirical thresholding categorized the continuous values from the reconstruction into the three values (representing tumor, lymphocyte and unoccupied grids) present in the original images.

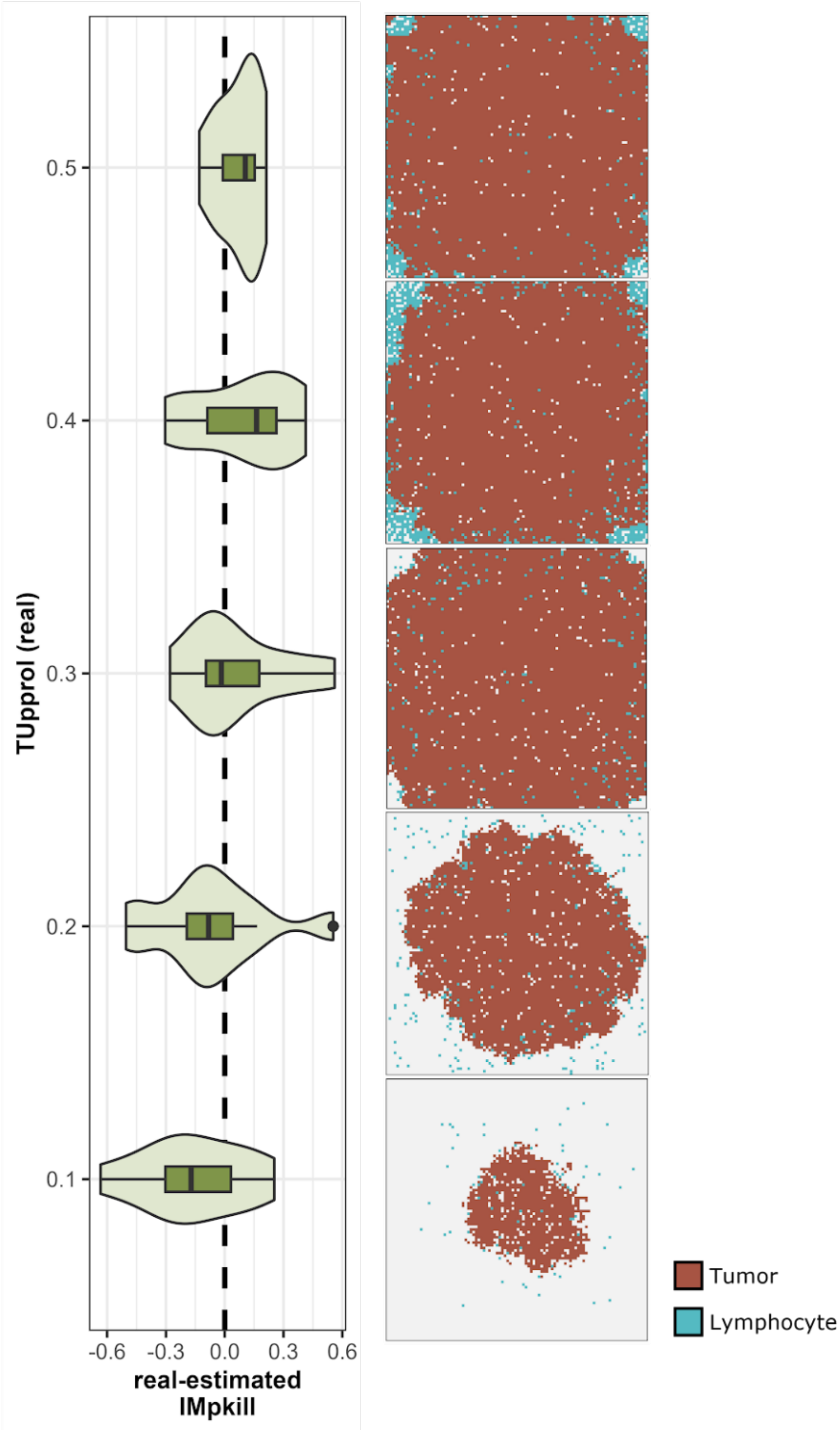

Supplementary Figure 2: Effect of TUpprol on differences between ground-truth and estimated IMpkill values. Violinplots represent the difference between ground-truth (real) and estimated IMpkill values, where positive values denote for instance an underestimation of IMpkill. Example spatial outputs are also provided for different TUpprol with IMpkill = 0.4 and IMrwalk = 1.

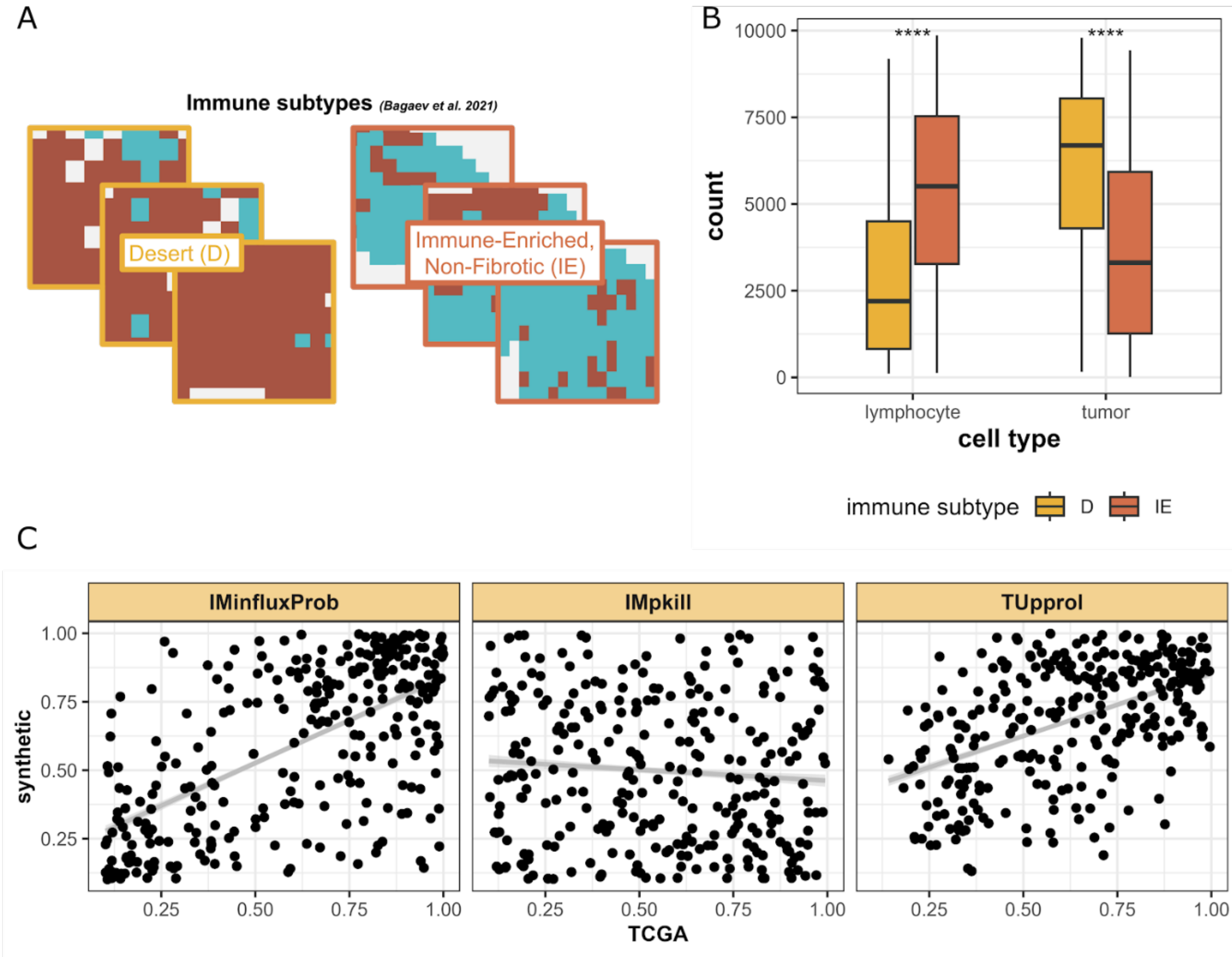

Supplementary Figure 3: TCGA omics-derived features. (A) Example patches for each immune subtype (desert, immune-enriched) **(B)** Cell counts of lymphocytes and tumor cells for each immune subtype, with corresponding hypothesis testing for significant differences in means. **(C)** Comparison of estimated IMinfluxProb, IMpkill and TUpprol parameter values with either the autoencoder trained on TCGA data or synthetic data.

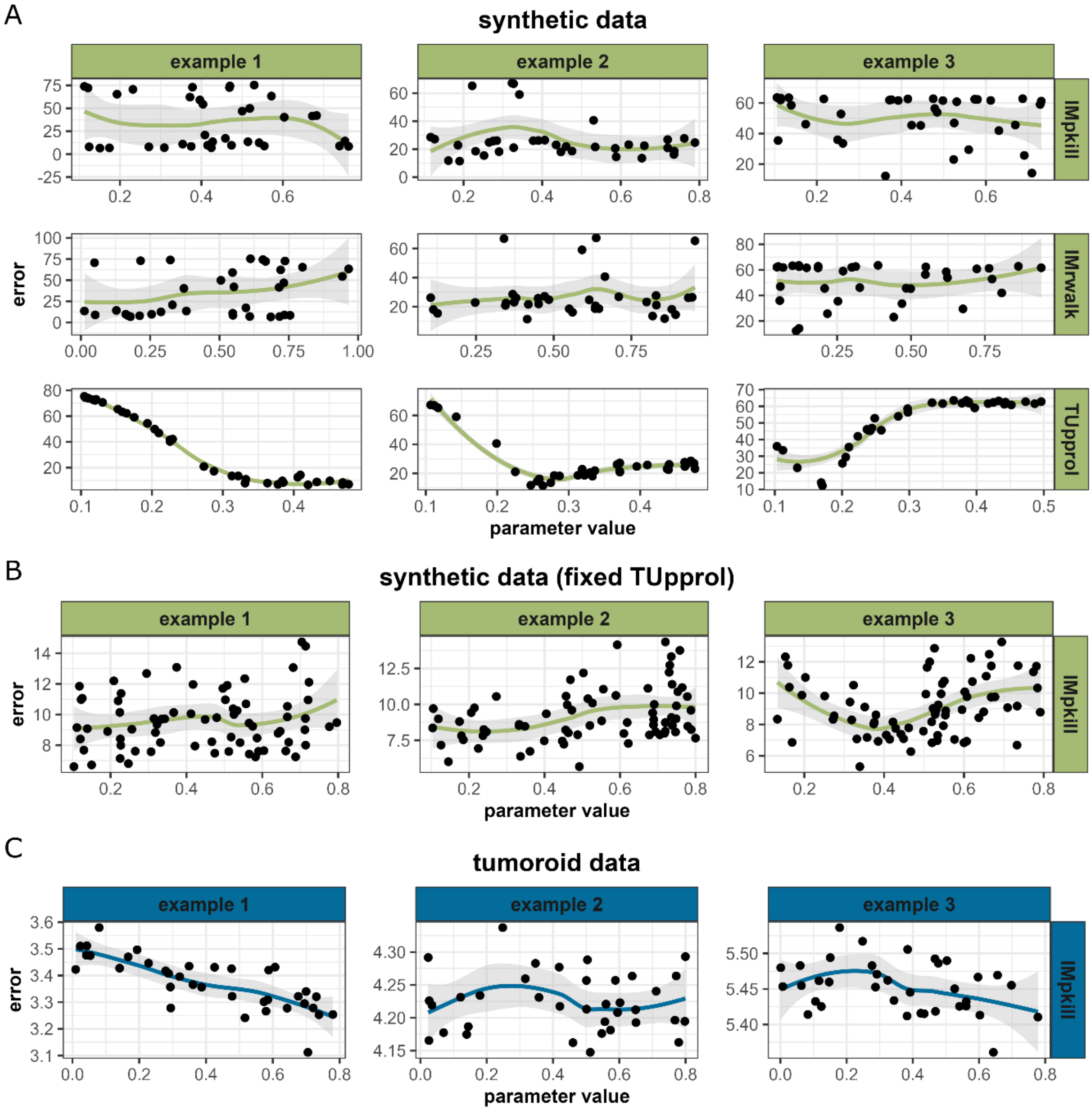

Supplementary Figure 4: Analysis of optimization error and parameter values. Representative examples of different trends found for the optimized parameters in the synthetic data **(A)**, synthetic data with fixed TUpprol **(B)** and tumoroid data **(C).** The data were retrieved from intermediate optimization trajectories and a local polynomial regression fitting was applied to highlight observed trends.

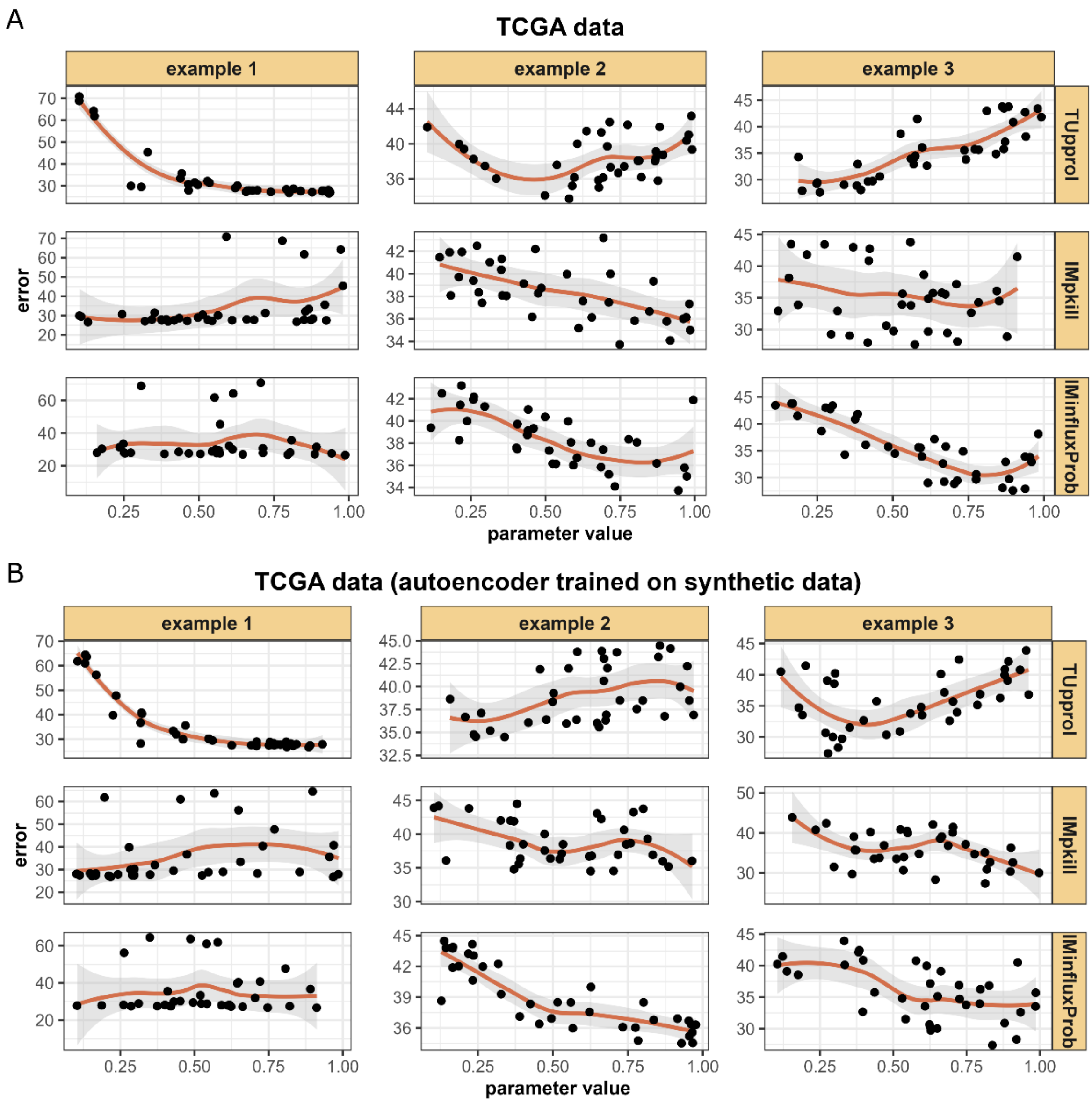

Supplementary Figure 5: Analysis of optimization error and parameter values. Representative examples of different trends found for the optimized parameters in the TCGA data **(A)** and TCGA images optimized with the autoencoder trained on synthetic data **(B)**. The same examples are visualized for both **(A)** and **(B)**, representing intermediate optimization values used to fit a local polynomial regression line.
